# Genomic evidence of genuine wild versus admixed olive populations evolving in the same natural environments in western Mediterranean Basin

**DOI:** 10.1371/journal.pone.0295043

**Published:** 2024-01-17

**Authors:** Lison Zunino, Philippe Cubry, Gautier Sarah, Pierre Mournet, Ahmed El Bakkali, Laila Aqbouch, Stéphanie Sidibé-Bocs, Evelyne Costes, Bouchaib Khadari

**Affiliations:** 1 AGAP Institut, University of Montpellier, CIRAD, INRAE, Institut Agro, Montpellier, France; 2 DIADE, University of Montpellier, CIRAD, IRD, Montpellier, France; 3 CIRAD, UMR AGAP Institut, Montpellier, France; 4 INRA, UR Amélioration des Plantes et Conservation des Ressources Phytogénétiques, Meknes, Morocco; 5 Conservatoire Botanique National Méditerranéen (CBNMed), UMR AGAP Institut, Montpellier, France; Institute of Mediterranean Forest Ecosystems of Athens, GREECE

## Abstract

Crop-to-wild gene flow is a mechanism process widely documented, both in plants and animals. This can have positive or negative impacts on the evolution of admixed populations in natural environments, yet the phenomenon is still misunderstood in long-lived woody species, contrary to short-lived crops. Wild olive *Olea europaea* L. occurs in the same eco-geographical range as domesticated olive, i.e. the Mediterranean Basin (MB). Moreover, it is an allogamous and anemophilous species whose seeds are disseminated by birds, i.e. factors that drive gene flow between crops and their wild relatives. Here we investigated the genetic structure of western MB wild olive populations in natural environments assuming a homogenous gene pool with limited impact of cultivated alleles, as previously suggested. We used a target sequencing method based on annotated genes from the Farga reference genome to analyze 27 western MB olive tree populations sampled in natural environments in France, Spain and Morocco. We also target sequenced cultivated olive tree accessions from the Worldwide Olive Germplasm Bank of Marrakech and Porquerolles and from an eastern MB wild olive tree population. We combined PCA, sNMF, pairwise *F*_ST_ and TreeMix and clearly identified genuine wild olive trees throughout their natural distribution range along a north-south gradient including, for the first time, in southern France. However, contrary to our assumption, we highlighted more admixed than genuine wild olive trees. Our results raise questions regarding the admixed population evolution pattern in this environment, which might be facilitated by crop-to-wild gene flow.

## 1 | Introduction

Gene flows between domesticated species and their wild relatives have been identified in several studies in animals [1–3] and plant species [4, 5]. This phenomenon is noted when cultivated genomic variants occur in unmanaged naturally occurring populations in natural environments. These admixed populations raise the question of the impact of gene flow on the evolution of natural populations. For instance, the introgression of new genetic diversity inside wild genomes can accelerate their evolution by increasing the frequency of favorable alleles, but can also be detrimental resulting in outbreeding depression, i.e. a loss of fitness in hybrids compared to their parents [6–8].

Cultivated to wild introgressions variants may be found in several plant species. Crop-to-wild gene flow occurs in maize and teosinte, its closest relatives, for instance, which has been found to lead to the acquisition of herbicide resistance in teosinte and, consequently, to high frequency of teosinte forms in maize fields [4]. In some perennial species such as apple, major introgressions with the spread of alleles from the cultivated gene pool to wild populations in Europe have been documented [9]. The resulting admixed populations showed higher fitness than wild apple trees [9, 10]. Gene flow from domesticated relatives has also been reported in natural chestnut and poplar populations. Admixed poplar populations have been found in France [11] and along the Danube River in natural environment [12], while the same scenario has been observed in chestnuts in Japan [13]. All these studies have proposed conservation measures for *in-situ* and *ex-situ* preservation of genuine wild populations by limiting gene flow, by replanting genuine wild genotypes far from domesticated forms or by protecting the connections of wild metapopulations which can breed and thereby protect themselves from random genetic deterioration [10, 12]. The evolutionary consequences of crop-to-wild gene flows in the natural environment and on wild populations are still misunderstood, especially in perennial species.

Olive tree, (*Olea europaea* L.) is an iconic perennial species from the Mediterranean Basin (MB) which can live thousands of years. Cultivated (*Olea europaea* var. *europaea*) and wild (*Olea europaea* var. *sylvestris*) forms coexist within the same Mediterranean distribution range [14, 15]. Wild olive trees have an ancient evolutionary history in the MB [16] indicated that three plastid lineages with a probable common ancestor dating from the Middle to Upper Pleistocene had diversified long before the Last Glaciation Maximum (26,500 to 19,000 BP [17]). They were subsequently impacted by glaciation, while some wild populations persisted in refugia [16, 18]. Olive lineages have been isolated in two distant areas, which could explain the current population genetic structure profile of wild olive trees. According to previous genetic studies, two main gene pools are identified, one in the eastern and another in the western/central MB [16, 19–21]. This eastern/western genetic differentiation is also found in other plants in the MB [22, 23]. Cultivated olive trees emerged with the domestication of olive trees around 6,000 years BP [14, 24, 25]. It is generally considered that the center of primary olive domestication, from wild progenitors, is located in the Middle East, near the border between Turkey and Syria [14, 16, 24].

It is currently impossible to distinguish between genuine wild, admixed and cultivated olive trees in the natural environment because of the absence of easily measurable discriminating morphological traits in the field. The use of geometric component of shape stone allows to distinguish the wild (round-shaped stone) from cultivated morphotype [26, 27]. However, numerous intermediate morphotypes have been found, ranging from elliptical to more tapered, reflecting the complex history and evolution processes related to human-associated migration [19, 21, 26, 28]. Genuine wild and admixed olive trees can only distinguish using genetic markers [19, 21, 28–31]. The genetic diversity of cultivated olive trees is close to eastern MB wild olive trees [20, 30, 31], hence making it difficult to study gene flow between eastern MB wild and cultivated accessions. Conversely, in western MB, the genetic diversity of wild olive trees growing in natural areas is clearly different from cultivated accessions [16, 21, 28], thereby enabling the identification of genuine wild olive trees as previously reported by [31] using allozyme markers. The genetic pattern observed in naturally occurring populations was little impacted by crop-to-wild gene flow [21, 28, 30]. The well-known genetic differentiation makes it a relevant model for investigating the genetic structure of populations in their natural environments and to infer potential gene flow between cultivated and wild olive.

Here, we investigated the genetic diversity of naturally occurring olive tree populations in the western MB. We assumed that the genetic pool of wild olive tree in the western Mediterranean area has not been impacted by introgressions from domesticated forms. We addressed the following questions: (1) What is the genetic structure of spontaneous olive trees in the western MB? (2) Are there genuine wild olive populations in this range? (3) Is there crop-to-wild gene flow in this region? We analyze genome-wide SNPs in olive trees from 27 natural sites ranging from southern France, Spain and Morocco. We included DNA from wild trees previously sampled in southern Turkey for the purpose of comparing diversity in these populations with the genetic pattern in the eastern MB wild gene pool [30]. We sought to identify crop-to-wild gene flow and patterns of admixtures using data of cultivated accessions from western MB from the Worldwide Olive Germplasm Bank of Marrakech (WOGBM) and Porquerolles obtained with the same sequencing strategy [32, 33].

## 2 | Methods

### 2.1 | Sampling of wild olive trees on a north-south gradient in the western Mediterranean Basin

**Fig 1:**
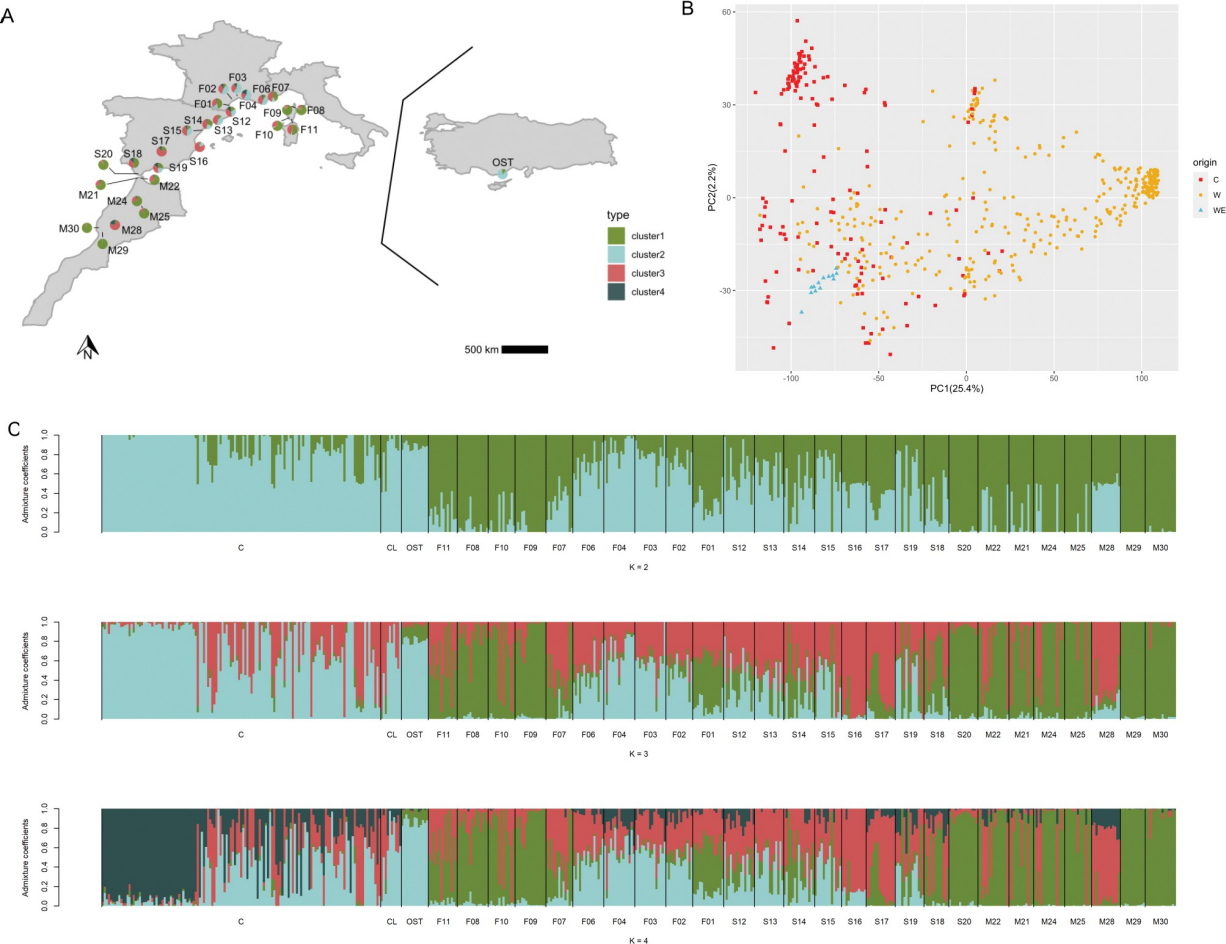
Result of the population structure analyses performed on the genome-wide SNPs diversity of natural populations of *O*. *europaea L*. collected in France (143), Spain (123), Morocco (96) and Turkey (13) and cultivated *O*. *europaea L*. from the western Mediterranean Basin (145), using 142,060 SNPs. (A) Geographical distribution of the populations and proportion of genetic cluster assigning. Pie chart at each location represents the fraction of individuals belonging to each genetic cluster as inferred by sNMF (K = 4). (B) PCA inferred with LEA. C: Cultivated, W: Western wild, WE: Eastern wild. (C) Genetic structure inferred by sNMF, each horizontal bar indicates individual assignment to a genetic cluster with K being the number of genetic clusters (K from 2 to 4).

Sampling of 27 assumed wild olive tree sites was conducted along a north-south gradient from southern France to southern Morocco in 2021 and early 2022. Sites were selected via the Conservatoire Botanique National Simethis database (http://simethis.eu) for southern France and northern Spain whereas for Corsica, central and southern Spain and Morocco, the delineation of wild populations was based on plastid polymorphism as reported by [21]. In addition to information from the Simethis database, we used environmental criteria to limit sampling of admixed olive populations and disregard olive orchards, agricultural and urban environments [34]. This resulted in the selection of 27 sites (Table 1; Fig 1A). At each of them, 13 to 15 individuals were sampled, representing a total of 400 sampled wild olive trees. At each site and for each tree, leaves were collected and immediately dried in silica gel for subsequent DNA extractions.

**Table 1:** Summary of the localisation of the natural olive populations sampled for the study.

Site	n	Country	Latitude	Longitude	Localisation		Source
F01	15	Continental France	42.9368	3.0126	Leucate	This study	
F02	15	Continental France	43.5407	3.3033	Lac des olivettes—Valhan	This study	
F03	15	Continental France	43.7706	3.7919	Cazevieille	This study	
F04	15	Continental France	43.8774	4.7328	Avignon	This study	
F06	15	Continental France	43.3826	6.3611	Plain des Maures—Gonfaron	This study	
F07	15	Continental France	43.6894	7.3029	MontBoron—Nice	This study	
F08	15	Corsica France	42.6538	9.0638	Ile Rousse	This study	
F09	15	Corsica France	42.4037	8.6989	Manso—Calvi	This study	
F10	15	Corsica France	41.7503	8.8688	Filitosa—Propriano	This study	
F11	15	Corsica France	41.3723	9.202	Bonifacio	This study	
S12	15	Spain	42.2355	3.2188	Roses	This study	
S13	15	Spain	41.4208	1.976	Barcelone	This study	
S14	15	Spain	41.0202	0.9348	L’Hospitalet del Infant	This study	
S15	15	Spain	40.3415	0.3858	Peníscola	This study	
S16	15	Spain	38.8029	0.1952	Xàbia	This study	
S17	15	Spain	38.3737	-3.507	Santa Elena	This study	
S18	15	Spain	37.2563	-6.2085	Aznalcar—Sevilla	This study	
S19	15	Spain	36.7656	-3.8496	Malaga	This study	
S20	15	Spain	36.0615	-5.6695	Tarifa	This study	
M21	15	Morocco	35.79	-5.9248	Cap Spartel	This study	
M22	15	Morocco	35.7828	-5.5153	Douar Dakchire	This study	
M23	13	Morocco	34.8685	-5.3526	Douar Nefzi	This study	
M24	15	Morocco	33.5341	-5.9082	Bouquachmir	This study	
M25	15	Morocco	33.0998	-5.5883	Moyen-Atlas—M’rirt	This study	
M28	15	Morocco	31.2104	-8.0398	Marrakech-Asni	This study	
M29	13	Morocco	30.6315	-9.3704	Ameskroud-Idmine	This study	
M30	15	Morocco	31.111	-9.6907	Agadir—Essaouira	This study	
OST	15	Turkey	36.11363	33.43209	Tisan	[16, 20, 28, 30, 31]	

*n*: sample size

### 2.2 | Reference set of cultivated and eastern wild accessions

In addition to the sample sites described above, leaves from 10 cultivated olive varieties were added to the sampling. These varieties were selected because of their significant presence in the French sampling area of natural populations [33], which can be a potential source of introgression. Fifteen individual wild olive trees from Turkey in the eastern MB [30] were also added to create a genetically distinct group which will be considered as an outgroup (Fig 1A). Moreover, 135 cultivated varieties from the WOGBM, representative of the genetic diversity of olive resources in the western MB [32] were considered as reference varieties to assess the introgressions from cultivated olive into wild populations. This last dataset was developed in a parallel study by our group that is focusing on cultivated olive (S2 Table). Overall, the experiment included 561 individuals.

### 2.3 | Bait design

The cultivated *Olea europaea* var. *europaea* (cv. Farga) Oe9 genome assembly [29] was used as a reference to design target sequencing probes. This genome is 1.38 Gb. Baits were designed according to the following parameters: place 80 bp probes with 0.5x tilling targeting the first 640 bp of each of the 55,595 annotated genes available. For each gene, 1 to 4 baits were designed depending on its length. After quality filtration, this resulted in a total set of 210,367 baits representing 55,452 unique loci and a captured length of 16.8 Mb. The probes were designed and synthesized by Daicel Arbor Biosciences, Ann Arbor, Michigan, USA. For this study, we only retained sequencing data targeted on assembled chromosomes. This subset represented 102,126 baits with a captured length of 8.2 Mb (S1 Table).

### 2.4 | Library preparation and sequencing

DNA was extracted from leaves using a mixed alkyl trimethylammonium bromide buffer (MATAB) and NucleoMag Plant Kit (Macherey-Nagel, Düren, Germany) as already described by [35] (S1 File). Individual genomic libraries for the NGS experiments were constructed with the NEBNext® Ultra™ II FS DNA Library Prep Kit (New England Biolabs, Ipswich, MA) with inputs ≤ 100 ng (S1 File). DNA was enzymatically sheared at an average 160 bp length before being tagged with the Unique Dual Index. Enrichment by capture was performed with biotinylated RNA probes (80 bp) as recommended by the provider using myBaits kits (Arbor Biosciences). A single dose of bait was used on a bulk of 48 normalized libraries. The sequencing was performed by MGX-Montpellier GenomiX on an Illumina® Novaseq^TM^ 6000 (Illumina Inc., San Diego, CA, USA) platform with an S4 flow cell. In addition to the target sequencing data set, we also sequenced four whole genomes (OES_E13_09, OES_F10_03, Picholine and Picholine Marocaine) to calculate the enrichment rate of the target sequencing method.

#### 2.4.1 | SNP calling

Raw sequencing reads were first trimmed with FastP version 0.20.1 [36]. The resulting data were then mapped on the reference genome Farga Oe9 genome assembly [29] using bwa-mem2 version 2.0 [37]. The mapped reads were sorted with samtools version 1.10 [38]. Only primary alignment, properly paired and unique reads were kept. Duplicates were removed using picard-tools version 2.24.0 [39]. From this clean alignment, GATK version 4.2.0.0 [40] was used for the SNP calling according to GATK4 best practices (https://gatk.broadinstitute.org/hc/en-us/sections/360007226651-Best-Practices-Workflows).

#### 2.4.2 | SNP filtering

After sequencing, we obtained 27,275,679 raw data. These were filtered with VCFtools version 0.1.16 [41]. The following filters were sequentially applied. With vcf-annotate, we first removed SNPs with a quality below 200 and clusters of 3 SNPs in 10 bases. VCFtools was used to remove indels, to only keep biallelic SNPs, to select SNPs with a minimum depth per site of 8 and a maximum mean depth per site of 400. Sites with >15% missing data were removed, then individuals with >20% missing data were also removed (S4 Table). After filtration, 35 individuals were removed (1 cultivated, 2 eastern wild plants and 32 western wild plants). Only sampling sites with at least 12 individuals were considered in this study. M23 had only 6 individuals left and was therefore removed from the data-set. Filtering was carried out to exclude positions with fixed heterozygosity (>85%) and the final filtering was done to keep at least one minor allele count per site. After all filtering steps, we obtained 142,060 SNPs in the final data set, these were located on all chromosomes (S3 Table).

### 2.5 | Genomic analysis

#### 2.5.1 | Genetic diversity and genetic structure

Genetic diversity measure was examined for each of sampled sites, considered as distinct populations. We calculated diversity measures as expected heterozygosity (*H*_E_), observed heterozygosity (*H*_O_) and inbreeding coefficient (*F*_IS_) using Hierfstat version 05–11 [42].

Pairwise between population genetic differentiations were estimated with Weir and Cockeman fixation index (*F*_ST_) using pairwise.WCfst function from the Hierfstat package version 05–11 [42]. Support values were calculated per locus, for each pair of population, based on bootstraps procedure (S5 Table).

Genetic structure analyses were conducted using sNMF and Principal Component Analysis (PCA) from the LEA package version 3.11.3 [43]. Both analyses were performed on the dataset. It included 135 individuals from the WOGBM, originating from Spain, France and Morocco according to their passport data, 10 cultivated samples from southern France, 362 western MB wild individuals and 13 eastern MB wild samples. For sNMF, five repetitions per clusters (K) considered, were performed with K ranging from 1 to 10.

#### 2.5.2 | Admixture assessment

Inference of the population history, with the admixture and split pattern were done using TreeMix version 1.13 [44]. This software constructs admixture graphs using allele frequencies of current genetic populations to infer a graph of all ancestral populations related to a common ancestor. For this analysis, we grouped cultivated olives in four different genetic groups, depending on their sNMF assignment to genetic ancestral clusters: C0, C1, C3 and C4 (S1 File; S6 Table). With these clusters, the 27 populations collected in western MB and the Turkish population, we did 100 TreeMix runs with a random SNP block size between 100 and 1000, from 1 to 10 migrations each when considering the M29 population as an outgroup. We inferred the optimum number of migrations with multiple linear models and the Evanno method implemented in the *OptM* package version 0.1.6 (S1 and S2 Figs) [45, 46]. TreeMix analysis was performed with 500 bootstrap replicates, which were used to build a consensus tree with Phylip version 3.697 [47]. We used BITE packages version 1.2.0008 [48] to display the trees.

## 3 | Results

In this study, we analyzed 520 individuals, including 145 cultivars from Spain, France and Morocco, representing the MB olive diversity of cultivated olive trees (S2 Table), a set of 362 wild trees from France, Spain and Morocco collected in 27 natural sites and 13 wild trees from southern Turkey.

### 3.1 | Target sequencing efficiency

The average enrichment rate in the target sequencing experiment was 34 times higher than expected with whole-genome sequencing (S7 Table). Moreover, for the bait on the chromosome annotated genes, 63.5% of the filtered SNPs (90,157) were on-target SNPs. The remaining was off-target SNPs. The on-target SNPs corresponded to sequences targeted by the baits. Conversely, the off-target corresponded to nonspecific and unintended sequences that can arise through sequencing (S7 Table).

### 3.2 | Genetic diversity and genetic structure

The average inbreeding coefficient (*F*_IS_) calculated on the different populations was on average 0. This is in accordance with the outcrossing mating system of olive tree. For 3 populations we detected *F*_IS_ values ranging from -0.086 to -0.101 (F03, F11, S13) while 3 other ones *F*_IS_ values ranging from -0.192 to-0.206 (S16, S17 and M28) (Table 2). All of these populations might be resulting from admixture event.

**Table 2:** Summary information of genetic diversity for sampling sites of naturally occurring olive trees in the western and eastern Mediterranean Basin.

	n	*H*_O_	*H*_E_	*F*_IS_
*F01*	15	0.142±0.195	0.141±0.178	-0.003±0.263
*F02*	13	0.147±0.199	0.144±0.180	-0.014±0.265
*F03*	15	0.145±0.213	0.130±0.177	-0.086±0.234
*F04*	15	0.134±0.202	0.124±0.174	-0.056±0.247
*F06*	15	0.148±0.202	0.142±0.180	-0.032±0.249
*F07*	13	0.154±0.218	0.141±0.184	-0.070±0.262
*F08*	15	0.127±0.197	0.121±0.173	-0.029±0.262
*F09*	15	0.121±0.194	0.118±0.172	-0.017±0.273
*F10*	13	0.135±0.205	0.126±0.176	-0.053±0.271
*F11*	14	0.147±0.222	0.129±0.181	-0.101±0.252
*S12*	15	0.158±0.214	0.146±0.184	-0.057±0.242
*S13*	14	0.155±0.225	0.136±0.182	-0.098±0.246
*S14*	15	0.154±0.204	0.149±0.184	-0.026±0.254
*S15*	13	0.148±0.205	0.142±0.182	-0.024±0.264
*S16*	12	0.187±0.274	0.144±0.187	-0.206±0.319
*S17*	14	0.168±0.264	0.131±0.187	-0.195±0.314
*S18*	12	0.147±0.204	0.141±0.182	-0.028±0.271
*S19*	14	0.140±0.199	0.140±0.184	0.001±0.280
*S20*	14	0.123±0.201	0.115±0.174	-0.043±0.269
*M21*	12	0.139±0.206	0.129±0.175	-0.051±0.262
*M22*	15	0.141±0.196	0.135±0.173	-0.030±0.246
*M24*	15	0.139±0.202	0.131±0.174	-0.045±0.247
*M25*	13	0.127±0.197	0.123±0.174	-0.020±0.270
*M28*	14	0.175±0.262	0.139±0.189	-0.192±0.295
*M29*	12	0.114±0.198	0.111±0.176	-0.018±0.307
*M30*	15	0.121±0.198	0.115±0.174	-0.031±0.268
*OST*	13	0.117±0.183	0.116±0.166	-0.007±0.271

n, number of genotypes; *H*_E_, expected heterozygosity; *H*_O_, observed heterozygosity; *F*_IS_, population level deviation from Hardy-Weinberg heterozygosity

**Fig 2:**
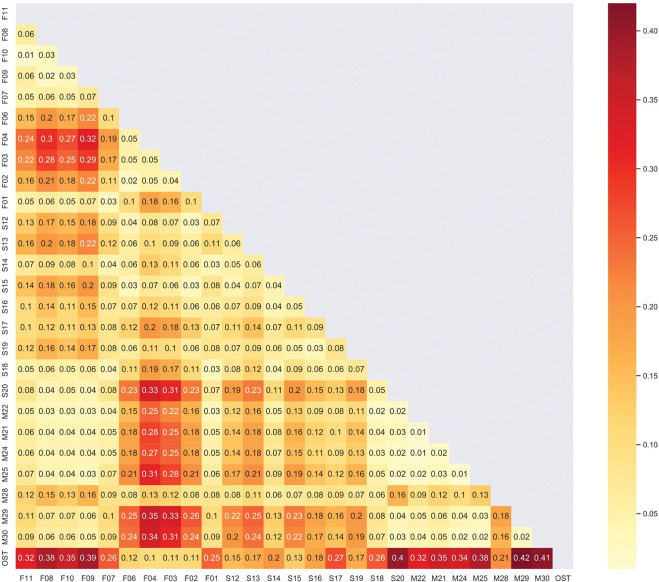
Heatmap of pairwise *F*_ST_ performed on the genome-wide SNPs diversity of 27 natural populations of *O*. *europaea* L. collected in western Mediterranean Basin in France (143), Spain (123), Morocco (96) and in the eastern Mediterranean Basin in Turkey (13).

The pairwise differentiation values between the studied populations (*F*_ST_) ranged from 0.01 to 0.42 (Fig 2). The Turkish eastern wild sampling site was the most genetically differentiated from the western MB sites, such as Corsica, F01 and F07 in Continental France, S14, S17, S18 and S20 in Spain, and all sites in Morocco (all above 0.2). Compared to the Turkish wild population, M29 from the southern limit of the olive distribution is the most differentiated population (*F*_ST_ = 0.42), while F04 from France was the least differentiated population (*F*_ST_ = 0.01). We revealed a high genetic differentiation between western MB wild populations and the eastern MB wild population.

**Fig 3:**
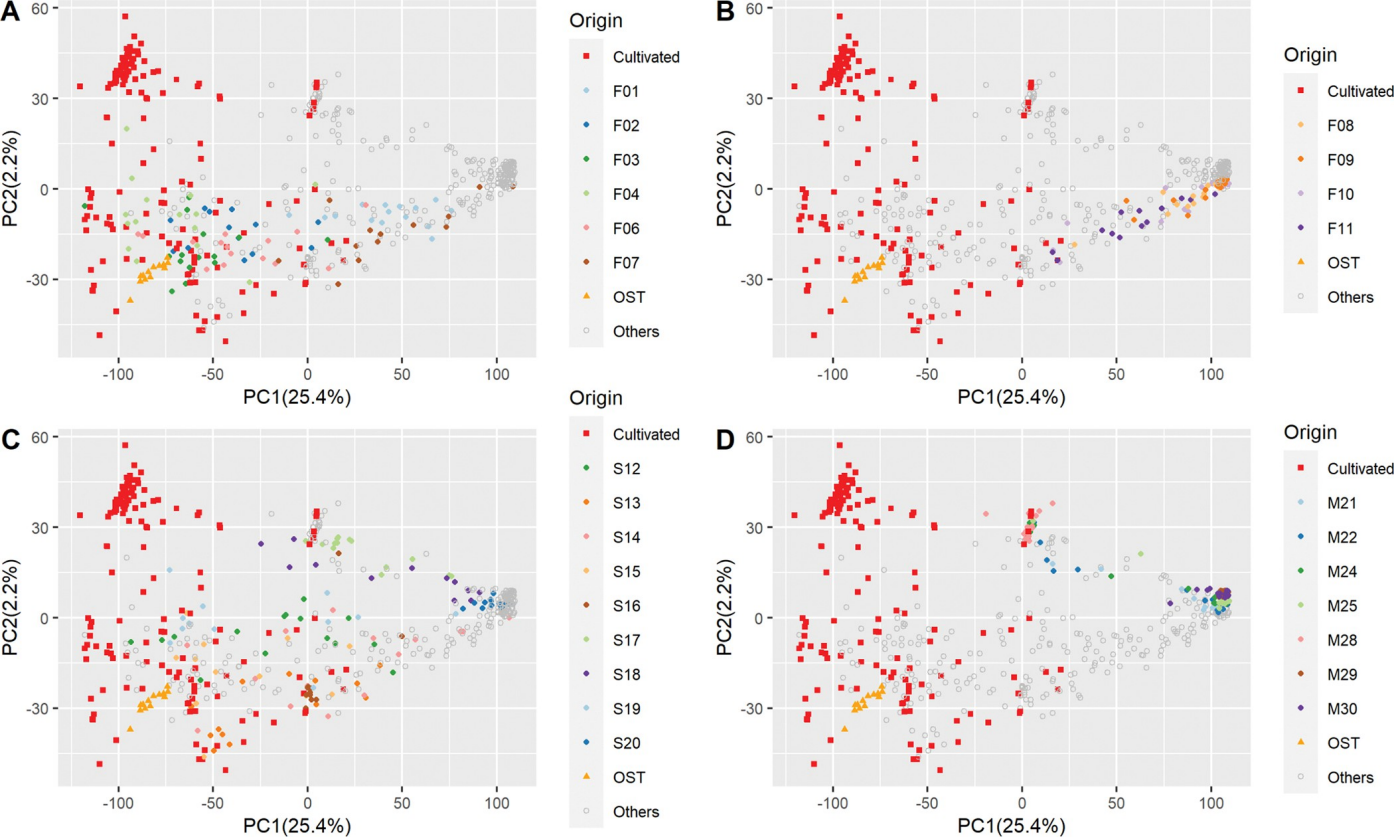
Detailed genetic structure of *O*. *europaea* L. populations using PCA analysis. Red boxes represent cultivated individuals, orange triangles represent Turkish population with the (A) French continental populations, (B) Corsican French populations, (C) Spain populations, (D) Moroccan populations.

In the principal component analysis (PCA), the first axis PC1 accounted for 25.4% of the variation and revealed an eastern-western genetic structure between western MB wild olive trees and eastern MB wild olive trees, with cultivated accessions mainly related to the eastern MB wild populations (Figs 1B and 3). On the first axis, we observed accessions from sampling sites in Corsica (F08 to F11, Fig 3B), S20, a large part of S18 from Spain (Fig 3C) and from all the sites in Morocco (Fig 3D), with the notable exception of M28, were clearly separated from the cultivated accessions and eastern MB wild accessions (Fig 3). All individuals collected in central southern France (F02, F03, F04 and F06; Fig 3A), one from Morocco (M28, Fig 3D) and some from north-central Spain (S12, S13, S15 and S16; Fig 3C) clustered with cultivated accessions as shown in the Figs 1B and 3 (left side of the PCA). This profile suggests admixture events. Several other individuals collected in eastern and western France (F01 and F07; Fig 3A), in Morocco (M21, M22, M24 and M25; Fig 3D) and in Spain (S14, S17, S18 and S19; Fig 3C) also exhibited a pattern of admixture with the cultivated accessions. The second axis, i.e. PC2, accounted for 2.2% of the observed variability, highlighting two subgroups within cultivated trees. The first subgroup includes cultivated genotypes mostly from Spanish varieties such as “Picual” and three Moroccan varieties including “Picholine Marocaine”. The other cultivated group included several varieties from Spain, Morocco and France (Fig 1B).

A similar pattern was supported by the sNMF analyses. According to the cross-entropy criterion, only K from 2 to 4 were considered suitable to explain the western MB natural olive tree genetic pattern (Fig 1C; S3 Fig). The wild Turkish olive population (OST) was assigned to a specific cluster from K = 2 to K = 4 (in blue) regardless of the admixture model examined, thereby supporting the existence of a structure between western MB and eastern MB natural populations. At K = 2, the cultivated and eastern MB olive trees collected in natural sites were assigned to cluster 1 (in blue) and the western MB wild natural olive trees in Morocco, Corsica, South Spain, France (F01 and F07 sites) were mainly assigned to cluster 2 (in green). Olive trees from the F02, F03, F04 and F06 sites in France were mainly assigned to cluster 1 (in blue). Trees collected from S12, S13, S15, S16, S17 S19 and M28 sites spread in the two clusters. At K = 3, these sampling sites were mostly assigned to cluster 3 (in red), particularly for individuals from populations M28, S17, and S16. With K = 4, a fourth cluster (in dark blue) was noted within the cultivated cluster, corresponding to the same first subgroup described in the PCA results above (Figs 1B and 3).

By combining three analyses (i.e. PCA, sNMF and pairwise *F*_ST_; Figs 1 and 3), particularly by considering the left and central part of the PCA (Figs 1B and 3) and the cluster 3 (in red; Fig 1C) from the sNMF analyses, we have several arguments strongly suggesting admixtures between natural olive trees and cultivated ones. It seems to be consistent with *F*_ST_ values, with much lower levels of differentiation between cultivated olive accessions and wild olive accessions (Fig 2). Accordingly, all olive trees mapped in the right side of the PCA (Figs 1B and 3) and assigned to the cluster (in green) regardless of the admixture model examined (Fig 1C) and considered as genuine wild.

### 3.3 | Inference of population admixture and gene flow

The tree inferred by TreeMix was ranked using M29 population. This population was chosen because all of the individuals collected in this site belong to the same cluster, referred as the western MB wild cluster (in green; Fig 1C). This genuinely western MB wild olive also appeared to be the most genetically distinct from eastern MB wild olive (*F*_ST_ = 0.42) and from the cultivated accessions (Fig 1).

**Fig 4:**
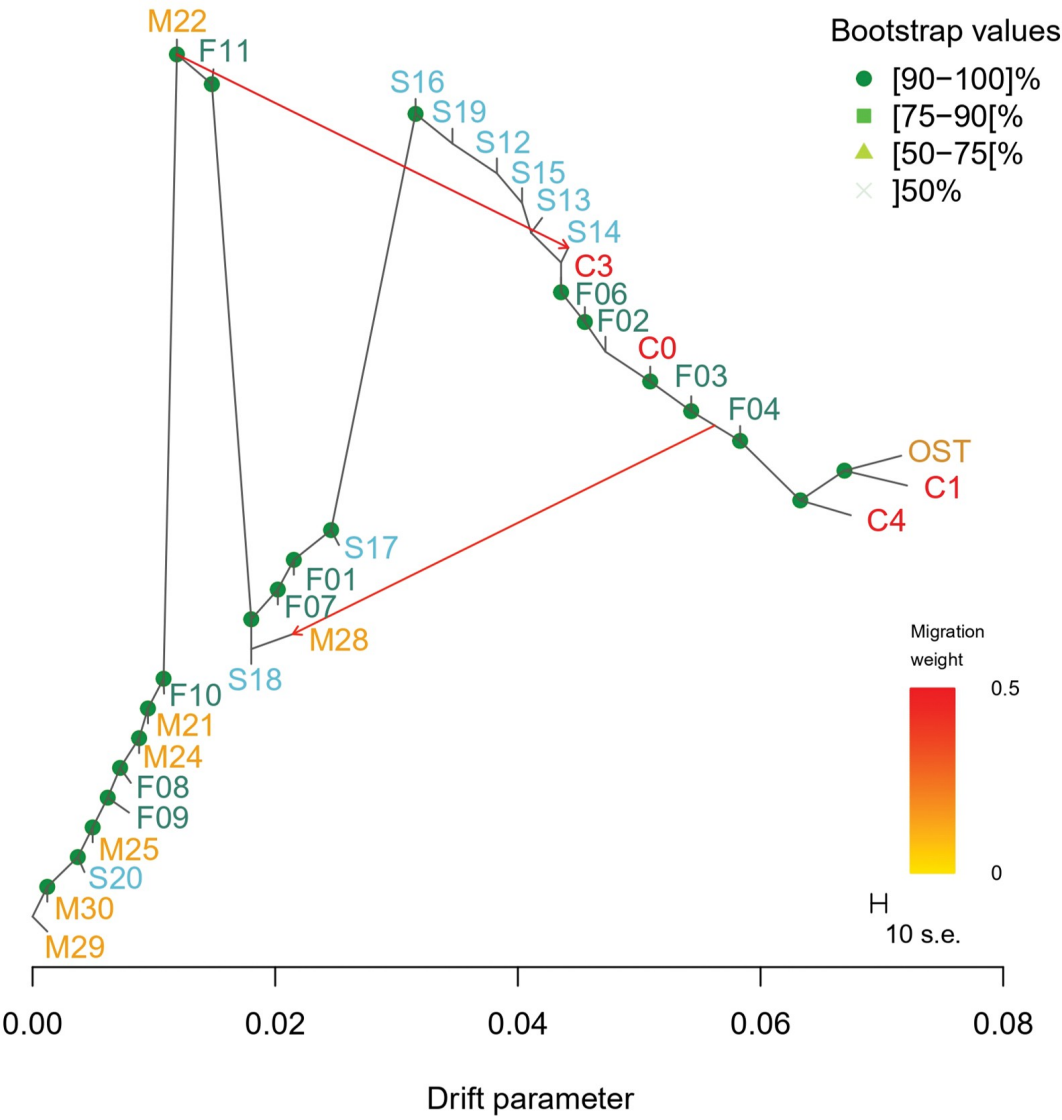
Tree inferred by TreeMix analysis on natural *O*. *europaea* L. populations from the western and eastern Mediterranean Basin and cultivated accessions. Genetic divergence is represented by the horizontal difference between populations. The vertical bars are only graphical representations and are not taken into account in the analysis. C0, C1, C3 and C4 are groups of cultivated accessions (S1 File).

The TreeMix analysis revealed the highest divergence between M29 and C3 (cultivated) and OST (Turkish population). The lowest divergence (below 0.015) from M29 were found for with almost all the Moroccan sites (except for M28), with S20, F08, F09, F10 and F11. A second group of sampling sites was found with a divergence from M29 of 0.017 to 0.024, including S18, M28, F07, F01 and S17. Accessions from sampling sites in Spain, except for S20, had a genetic divergence of >0.032 from M29 and were closer to cultivated groups (<0.012 genetic divergence between S16 and C3). Accessions from the French F06, F02, F03 and F04 sites were found to be grouped with the cultivated clusters C3 and C0. TreeMix inferred a low divergence between C4, C1 and OST (around 0.005). Two gene flow events were inferred (S1 and S2 Figs), with the first one being from cultivated and French populations from the center to M28, with a weight of (w = 0.425). The second one was from M22 to northern Spain sites (w = 0.485) (Fig 4).

## 4 | Discussion

Wild olive molecular identification and characterization are important to assess the genetic diversity of this species, in addition, to evolutionary history and to investigate local adaptation. This is particularly important for wild relative crops such as olive, where wild olives and cultivated olives coexist in the same area [14, 15] and cannot be clearly distinguish with morphological traits [26, 27] but only using genetic markers [19, 21, 28–31]. As crop-to-wild gene flows likely occur, deciphering their genetic relationships can help to understand the impact of crop on natural populations, their demographic histories and to explore new sources of genetic diversity. In this study, our aim was to identify genuine wild olive populations by investigating the genetic structure and diversity patterns of olive trees evolving in the natural environment in the western MB. We sampled allegedly wild olive trees according to past study [21] over a large geographic area in natural environment within the western MB. We hypothesis that all these populations are genuine wild olives. In addition, we included eastern MB genuine wild olive trees and cultivated olive trees. We analyzed this large panel of population using SNPs from target sequencing. This if the first time target sequencing has been used to study genomic variation in olive tree—it enabled a genome scan of many individuals while accessing more than 140,000 SNPs distributed throughout the genome. This represents a major advance over previous methods in similar studies using SSR-based molecular analyses [21, 28] or SNPs analyses from RNAseq [30]. We documented the presence of genuine wild olive tree populations in the western MB, in southern France, Spain and Morocco. However, contrary to our assumption, our analysis suggested that admixed populations are more frequent than genuine ones.

### 4.1 | Genetic variation patterns in natural and cultivated olive trees highlight the persistence of genuine wild olive populations in the western Mediterranean Basin

Based on a large set of SNP markers, located all over the genome, we identify a very strong genetic differentiation between the natural populations collected in the western MB and those in the eastern MB. This confirmed the results of previous studies using microsatellite markers highlighted two distinct gene pools in the eastern and western MB. In these previous studies, the western/central MB gene pool clustered wild populations from Greece to Morocco [21, 28] while the eastern MB gene pool clustered wild populations from Greece to the Levant. The eastern MB gene pool was represented here by one Turkish population (OST), i.e. a genuine wild population previously revealed using plastid DNA polymorphism [16, 21], SSR markers [28] and SNPs from RNAseq [30]. Our results reflects the typical long evolutionary history of Mediterranean species as described by [49].

Our study confirmed that the wild eastern MB olive population from Turkey is very closely related to cultivated olives, as shown by different approaches (PCA, sNMF, *F*_ST_ and TreeMix). This finding is consistent with the olive domestication history which was likely the results of genetic selection of eastern MB wild trees [16, 28, 30]. Moreover, we combined several approaches, i.e. PCA, sNMF, pairwise *F*_ST_ estimation and inference of splits in mixture in populations and clearly identified a genetic group of olive trees strongly differentiated from the eastern wild and cultivated olive as previously found by [30] which probably constitute genuine wild olive trees. Finally, within cultivated accessions, we identified two genetic groups. The first one similar to OST and which might be composed of varieties likely issued from two processes, primary selection in the east and secondary diversification in the central and western Mediterranean areas as proposed by Khadari & El Bakkali (2018). The second group essentially consisted of Spanish and Moroccan varieties that were highly differentiated from OST and which might be composed of western MB cultivated olives through a secondary diversification process mainly via selection involving crossing between ancient varieties such as Gordal Sevillana and Lechin de Granada, as shown by [19].

### 4.2 | Wild versus admixed olive trees: An evolutionary history impacted by domestication

As discussed above, we identified genuine wild olive trees in the western Mediterranean basin in Spain, Corsica (France) and Morocco as already shown by [21]. The genetic diversity observed in Corsican and Moroccan populations were very close suggesting a common ancestral history for these populations. We also discovered, for the first time, the presence of genuine wild olive trees in continental France at Mont-Boron (eastern-south; F07) and near Leucate (western-south; F01). The abundance of wild olives trees in the western MB has been well characterized [16, 20, 28, 30, 31]. However, populations initially characterized as genuine wilds according to plastid DNA polymorphism and SSR markers, for instance, in Spain [21] were found admixed in our study. These sites were in habitats considered to be little or not at all impacted by human activities, e.g. in natural reserves, remote from urban centres or areas with olive orchards. Even within some sites, from a genetic viewpoint, several individuals were considered genetically to be wild, while others were very admixed with cultivated olives. This high genetic admixture intensity was unexpected in the western MB based on previous studies [16, 21, 28, 30, 50]. This differing results compared to past study [21, 28] may arise from variations in the sampling distribution area and the size of the samples at each site, particularly with the presence of heterogeneous populations, including admixed and wild individuals. The SSRs should be able to detect a similar pattern of admixture. Our findings have provided new insights into the evolutionary history of olive trees in natural western MB habitat.

### 4.3 | What factors could influence the prevalence of admixed populations in natural environments?

We obtained clear evidence in this study on the substantial presence of admixed populations within the natural olive populations which were previously reported to be little impacted by crop-to-wild gene flow [21, 28]. Recent phylogenomic and population structure investigations revealed genetic admixtures during olive domestication thus highlighting the impact of domesticated alleles on two wild olive trees for the western MB [29]. However, these authors analyzed very limited sampling of wild olive trees (7 olive trees from the western MB), while in our investigations, we analyzed 362 trees sampled from 27 natural sites in Spain, Morocco and France—this sampling covered the olive natural distribution range in the western MB. Through TreeMix analysis and comparing genomic variation comparison in these 362 olive trees to the genuine Turkish natural population and to cultivated olive trees, we, therefore, were able to depict gene flow between wild and cultivated olives (see Fig 4). The observed patterns highlighted the two kinds of gene flow events: from cultivated to wild populations in Morocco, France and Spain and from wild to cultivated or already admixed populations in Spain.

These gene flows could have been driven by several factors. First, the mating system of olive, which, is allogamous, pollination mostly depends on wind (anemophilous pollination) and seed dissemination relies on birds (zoochorous dissemination). These forms of dissemination may occur over long geographic distances (>50 km) [51]. Second, cultivated and wild olive trees share the same climatic and ecological niches, the geographic proximity between them increases the possibility of gene flow and events of admixture [50]. Third, there could be cultivated versus wild olive tree pollen competition: monocultures and single-varietal olive orchards are responsible for broad dissemination of pollen from orchards (thousands of trees), whereas wild populations are often composed of few individuals. Wild olive pollen is thus less abundant. Fourth, gene flow between cultivated and wild olive trees may increase genetic diversity in admixed populations. Associated new variants or combination might be better adapted to the local environment, promoting an acceleration of local adaptation of a species to an environment is a recognized evolutionary force explaining the occurrence of admixed populations [8]. Evolutionary factors such as allogamy [52] and cultivated versus wild olive tree pollen competition (see above) may not be sufficient to explain the large frequency of admixed populations versus genuine wild populations. Here we assume that admixed olive trees could have a better adaptive potential to their natural environment, as this has been previously demonstrated in several short-lived and annual crops [3, 4, 10]. This assumption is supported by the findings of genetic investigations on natural olive trees in Australia [53]. These authors hypothesized that hybridization between two introduced *Olea* species, *Olea europaea* subsp. *europaea* and *Olea europaea* subsp. *cuspidata*, overcame the lack of diversity after their introduction bottleneck, thereby facilitating their establishment. Strikingly, to our knowledge, this assumption has yet to be investigated in long-lived woody plants such as olive trees, even though knowledge on the impact of admixture on the evolution of natural populations could help guide appropriate conservation strategies in forest areas and other natural ecosystems.

### 4.4 | Consequences of extensive hybridization of wild olive via domesticated olive introgression and conservation recommendations

The future of genuine wild genotypes might be threatened by the gene flow we highlighted here. For instance, extensive gene flow could ultimately lead to complete replacement of wild populations by admixed genotype [8]. However, in global change context, this gene flow could enhance adaptation to a changing environment. Our study offers new opportunities for more in-depth studies on this introgression process. We identified three different compartments, i.e. a genuine wild olive compartment, a cultivated one and an admixed one, that could be study to address this long-standing question. Conservation policies on wild olive trees should take into account the risk of introgression from cultivated alleles and by the impact of climate change. The naturally occurring olive trees sampled here were positioned on a north-south gradient with different environmental conditions, which could facilitate studies on their potential local adaptation to changing climatic conditions. Our study finding may provide a basis for designing new conservation measures to protect genuine wild genotypes, *in-situ* and *ex-situ*, including repositories of wild genetic diversity not impacted by artificial selection.

## 5 | Conclusion

In this study we assessed the genetic structure of natural olive populations from the western Mediterranean Basin. We confirmed that the western MB genuine wild olive is genetically well differentiated from eastern MB wild olive as well as cultivated forms. We detected its presence in France, Spain and Morocco. We also found many admixed populations resulting from strong crop-to-wild gene flow. The presence of admixed olive populations in the same distribution area as genuine wild populations raises questions on the reasons for their predominance in the natural environment and on designing conservation strategies for both compartments. Finally, the two genetic patterns revealed by our investigations could be considered as a suitable model for investigating two core questions, the first on the admixture nature, i.e. what domesticated genomic alleles/regions would be suitable for introgression in wild genomes? The second question is related to local adaptation: are wild better locally adapted than admixed olive trees?

## Supporting information

S1 File Supplementary methods. Supplementary methods for the DNA extraction, Library preparation, assignment to create subgroup inside cultivated.
(DOCX)

S1 Fig Multiple linear models of possible migration events between 27 naturally occurring populations, eastern wild Mediterranean population and cultivated accessions of olives using TreeMix. One hundred TreeMix runs were done with a random SNPs block size between 100 and 1000, from 1 to 10 migrations each with M29 as outgroup. The analysis was performed with OptM package.
(TIF)

S2 Fig Evanno test of possible migration events between 27 naturally occurring populations, eastern wild Mediterranean population and cultivated accessions of olives using TreeMix. One hundred TreeMix runs were done with a random SNPs block size between 100 and 1000, from 1 to 10 migrations each with M29 as outgroup. The analysis was performed with OptM package.
(TIF)

S3 Fig Cross-entropy criterion inferred by sNMF analyze performed on the genome-wide SNPs diversity of natural populations of *O*. *europaea* L. collected in France (143), Spain (123), Morocco (96) and Turkey (13) and cultivated *O*. *europaea* L. from the western Mediterranean Basin (145), using 142,060 SNPs. (TIF)

S1 Table List of the baits used in the study. The baits were designed based on annotated gene of *Olea europaea* var. *europaea* (cv. Farga) Oe9 genome assembly (Julca et al. 2020).
(XLSX)

S2 Table Summary information of the sequenced cultivated accessions of *O*. *europaea* L. used in the study. (DOCX)

S3 Table List of filters and remaining SNPs for each on genomic data of target sequencing of 561 genotypes of *O*. *europaea* L. (DOCX)

S4 Table List of individuals of *O*. *europaea* L. removed from genomic data set because of missing data >0.2. (DOCX)

S5 Table Results of bootstraping over loci of pairwise *F*_ST_ performed on the genome-wide SNPs diversity of natural populations of *O*. *europaea* L. collected in France (143), Spain (123), Morocco (96) and Turkey (13) using 142,060 SNPs. The upper limit ci is the upper part and the lower limit ci in the bottom part of the matrix.
(XLSX)

S6 Table List of cultivated accessions of *O*. *europaea* L. and their assignment to genetic cluster (C0, C1, C3 or C4) according to sNMF assignation at K = 4. (DOCX)

S7 Table Descriptive data of depth and enrichment rate of *O*. *europaea* L. using target sequencing method versus whole genome sequencing method. (DOCX)
