# Supplementary material for: Genomic evidence of genuine wild versus admixed olive populations evolving in the same natural environments in western Mediterranean Basin
Source: PLoS One. 2024 Jan 17;19(1):e0295043. doi: 10.1371/journal.pone.0295043 (PMC10793901; doi:10.1371/journal.pone.0295043)
Supplement: S1 File — Supplementary methods for the DNA extraction, Library preparation, assignment to create subgroup inside cultivated. (DOCX) [file pone.0295043.s001.docx]

## S1 File. Supplementary methods

### DNA extraction:

DNA extraction was performed on leaves dried after a minimum of 10 days in silica gel. 25 mg of dried samples were sorted in a Deepwell 96 well plate (Corning 1.1mL Deepwell ThermoFisher Scientific) with beads. The plate was grinded using GenoGrinder (SPEX SamplePrep). The DNA was extracted using a MATAB buffer-based extraction method. Genomic DNA was quantified with Fluoroskan (Thermo fisher Scientific Inc). The quality was checked using agarose gel electrophoresis and Nanoquant plate (Tecan)

### Library preparation:

The input DNA was fragmented for 10 minutes with fragmentase enzymes. The fragmentation size was checked with Tapestation with a mean average size of 160 bp. The adaptors were ligated then the samples were purified with AMPure beads (Beckman Coulter, Inc) at 0.8X to do a low and high size selection of 200 bp. Unique Dual Index (NEBNext Multiplex oligos for Illumina) were added to the adaptor-ligated DNA before doing an enrichment PCR as required for NovaSeq experiments. Libraries were amplified for 11 cycles and cleaned with AMPure beads at 0.9X. The library quality was checked with Tapestation. Each library was quantified by qPCR on a LightCycler (Roche Molecular Systems, Inc) before being mixed together on a 48 libraries equimolarity pool. Hybridization capture for targeted NGS protocol is applied on each pool. The baits are added in a pool before hybridizing with beads for at least 16 hours at 60°C. The beads are then washed and the Bait-target hybrids are amplified by PCR for 12 cycles. The reaction is purified with AMPure beads. The captured library quality was verified by Tapestation.

Each capture-pool was quantified by qPCR on LightCycler before being grouped in one final pool of 384 libraries based on equimolarity.

### Cluster assignment to create subgroup inside cultivated:

Using sNMF from LEA packages V3.11.3 (Frichot and François 2015), we did structure analyses of the subgroup of cultivated (cultivated from west, including varieties from Spain, Morocco and France). Five runs were performed with K from 1 to 10. According to the cross-entropy, only K of 4 was represented in the results. Using this, we did an assignment of each variety to a cluster, if the variety was assigned to a cluster more than 70%. If the individual is not assigned to any cluster with these parameters, then it is assigned to an admixed cluster, C0. The C2 group was composed only with 3 individuals, which are not sufficient to a TreeMix analysis, we decided to put them in the C0 group and remove C2.

References :

**Frichot E**, **François O**. **2015**. LEA: An R package for landscape and ecological association studies. *Methods in ecology and evolution / British Ecological Society* **6**: 925–929.
