# Supplementary figures and images for: Genomic evidence of genuine wild versus admixed olive populations evolving in the same natural environments in western Mediterranean Basin
Source: PLoS One. 2024 Jan 17;19(1):e0295043. doi: 10.1371/journal.pone.0295043 (PMC10793901; doi:10.1371/journal.pone.0295043)

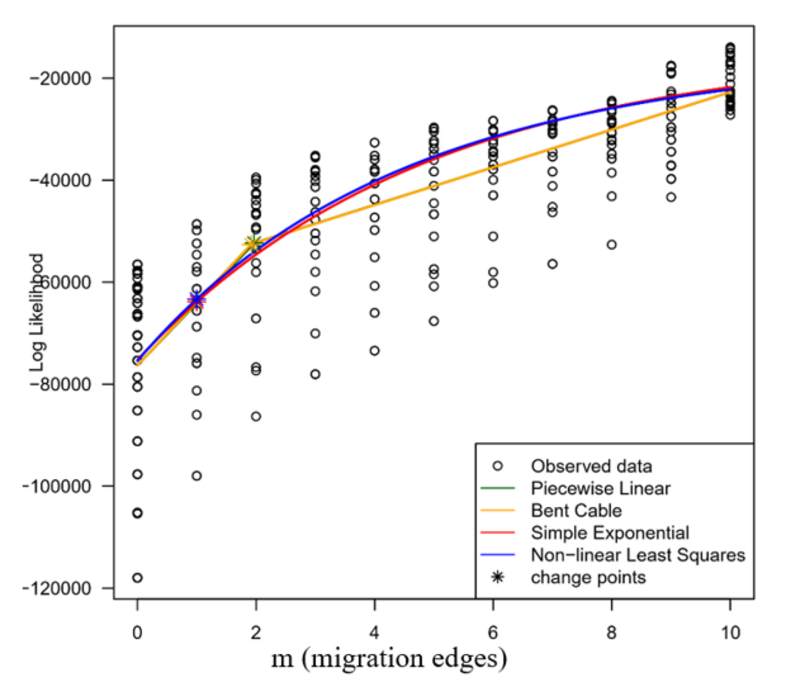

Supplement: S1 Fig — One hundred TreeMix runs were done with a random SNPs block size between 100 and 1000, from 1 to 10 migrations each with M29 as outgroup. The analysis was performed with OptM package. (TIF) [file pone.0295043.s002.tif]

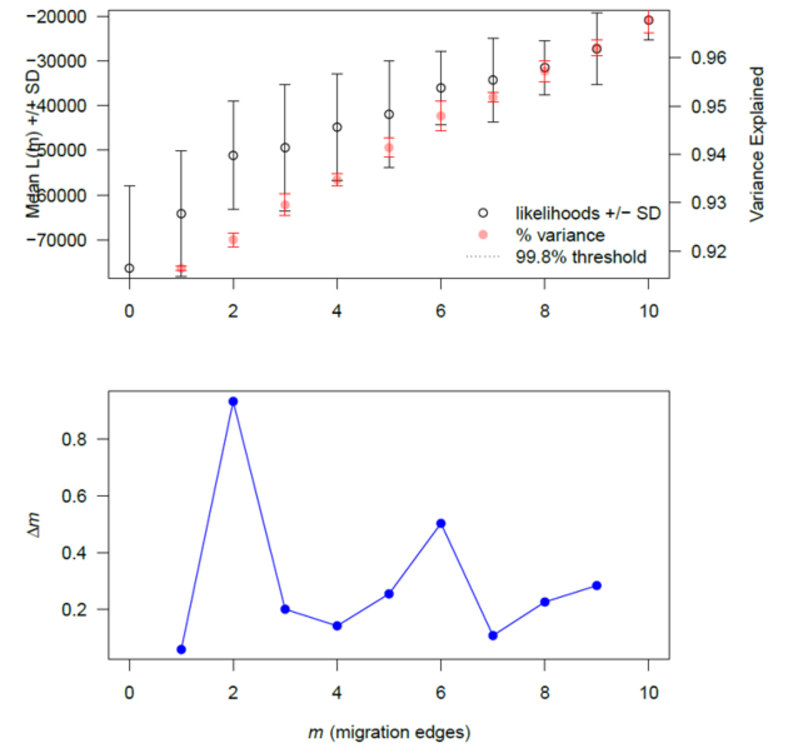

Supplement: S2 Fig — One hundred TreeMix runs were done with a random SNPs block size between 100 and 1000, from 1 to 10 migrations each with M29 as outgroup. The analysis was performed with OptM package. (TIF) [file pone.0295043.s003.tif]

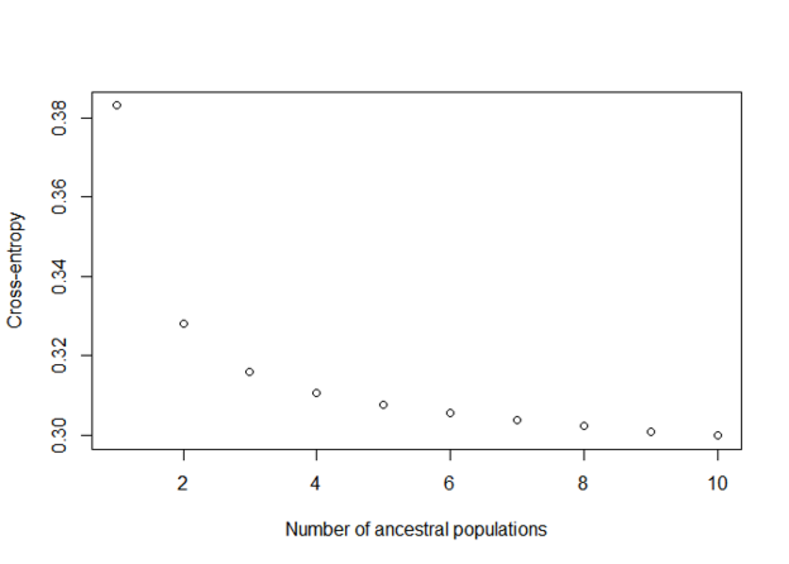

Supplement: S3 Fig — (TIF) [file pone.0295043.s004.tif]
