# Supplementary material for: Genomic evidence of genuine wild versus admixed olive populations evolving in the same natural environments in western Mediterranean Basin
Source: PLoS One. 2024 Jan 17;19(1):e0295043. doi: 10.1371/journal.pone.0295043 (PMC10793901; doi:10.1371/journal.pone.0295043)
Supplement: S2 Table — (DOCX) [file pone.0295043.s006.docx]

**S2 Table**. **Summary information of the sequenced cultivated accessions of *O. europaea* L. used in the study**

| **Sample ID** | **Code** | **Accession origine** | | **Cultivar origine** | **Source** |
| --- | --- | --- | --- | --- | --- |
| Unkown-VS2-545 | MAR00545 | | Morocco | Morocco | World Olive Germplasm Bank of Marrakech |
| Acebuchera | MAR00215 | | Spain | Spain | World Olive Germplasm Bank of Marrakech |
| Aglandau | MAR00187 | | France | France | World Olive Germplasm Bank of Marrakech |
| Alameno Blanco | MAR00216 | | Spain | Spain | World Olive Germplasm Bank of Marrakech |
| Alameno de Montilla | MAR00218 | | Spain | Spain | World Olive Germplasm Bank of Marrakech |
| Amargoso | MAR00219 | | Spain | Spain | World Olive Germplasm Bank of Marrakech |
| Arbequina | MAR00220 | | Spain | Spain | World Olive Germplasm Bank of Marrakech |
| Azul | MAR00221 | | Spain | Spain | World Olive Germplasm Bank of Marrakech |
| Berri Meslal-397 | MAR00397 | | Morocco | Morocco | World Olive Germplasm Bank of Marrakech |
| Berri Meslal-532 | MAR00532 | | Morocco | Morocco | World Olive Germplasm Bank of Marrakech |
| Bical | MAR00333 | | Spain | Spain | World Olive Germplasm Bank of Marrakech |
| Blanqueta | MAR00222 | | Spain | Spain | World Olive Germplasm Bank of Marrakech |
| Bolvino | MAR00223 | | Spain | Spain | World Olive Germplasm Bank of Marrakech |
| Borriolenca | MAR00334 | | Spain | Spain | World Olive Germplasm Bank of Marrakech |
| Bouchouika | MAR00394 | | Morocco | Morocco | World Olive Germplasm Bank of Marrakech |
| Bouteillan | MAR00189 | | France | France | World Olive Germplasm Bank of Marrakech |
| Callosina | MAR00391 | | Morocco | Spain | World Olive Germplasm Bank of Marrakech |
| Canivano Negro | MAR00224 | | Spain | Spain | World Olive Germplasm Bank of Marrakech |
| Carrasqueno de Jumilla | MAR00226 | | Spain | Spain | World Olive Germplasm Bank of Marrakech |
| Carrasquillo | MAR00335 | | Spain | Spain | World Olive Germplasm Bank of Marrakech |
| Cayon | MAR00191 | | France | France | World Olive Germplasm Bank of Marrakech |
| Cerezuela | MAR00349 | | Spain | Spain | World Olive Germplasm Bank of Marrakech |
| Changlot Real | MAR00227 | | Spain | Spain | World Olive Germplasm Bank of Marrakech |
| Chorruo | MAR00229 | | Spain | Spain | World Olive Germplasm Bank of Marrakech |
| Cirujal | MAR0043 | | Italy | Spain | World Olive Germplasm Bank of Marrakech |
| Corbella | MAR00230 | | Spain | Spain | World Olive Germplasm Bank of Marrakech |
| Cornezuelo de Jaen | MAR00231 | | Spain | Spain | World Olive Germplasm Bank of Marrakech |
| Cornicabra | MAR00232 | | Spain | Spain | World Olive Germplasm Bank of Marrakech |
| Cucca | MAR0032 | | Spain | Spain | World Olive Germplasm Bank of Marrakech |
| Dolce di Rossano | MAR0011 | | Spain | Spain | World Olive Germplasm Bank of Marrakech |
| Dressi | MAR00286 | | Spain | Spain | World Olive Germplasm Bank of Marrakech |
| Dulzal | MAR00233 | | Spain | Spain | World Olive Germplasm Bank of Marrakech |
| El Lewa | MAR00494 | | Spain | Spain | World Olive Germplasm Bank of Marrakech |
| Empeltre | MAR00234 | | Spain | Spain | World Olive Germplasm Bank of Marrakech |
| Enagua de Arenas | MAR00336 | | Spain | Spain | World Olive Germplasm Bank of Marrakech |
| Escarabajuelo de Posadas | MAR00235 | | Spain | Spain | World Olive Germplasm Bank of Marrakech |
| Escarabajuelo de Úbeda | MAR00236 | | Spain | Spain | World Olive Germplasm Bank of Marrakech |
| Farga | MAR00338 | | Spain | Spain | World Olive Germplasm Bank of Marrakech |
| Frantoio | MAR0039 | | Spain | Spain | World Olive Germplasm Bank of Marrakech |
| Fulla de Salze | MAR00339 | | Spain | Spain | World Olive Germplasm Bank of Marrakech |
| Gentile di chieti | MAR0015 | | Spain | Spain | World Olive Germplasm Bank of Marrakech |
| Gordal Sevillana | MAR00108 | | Italy | Spain | World Olive Germplasm Bank of Marrakech |
| Gordal de Granada | MAR00238 | | Spain | Spain | World Olive Germplasm Bank of Marrakech |
| Grappolo | MAR0041 | | Spain | Spain | World Olive Germplasm Bank of Marrakech |
| Grossane-194 | MAR00194 | | France | France | World Olive Germplasm Bank of Marrakech |
| Habichuelero de Grazalema | MAR00239 | | Spain | Spain | World Olive Germplasm Bank of Marrakech |
| Hojiblanca | MAR00240 | | Spain | Spain | World Olive Germplasm Bank of Marrakech |
| Idleb | MAR00591 | | Spain | Spain | World Olive Germplasm Bank of Marrakech |
| Jabaluna | MAR00241 | | Spain | Spain | World Olive Germplasm Bank of Marrakech |
| Jaropo | MAR00242 | | Spain | Spain | World Olive Germplasm Bank of Marrakech |
| Khashabi-631 | MAR00631 | | Spain | Spain | World Olive Germplasm Bank of Marrakech |
| Lastrino | MAR0023 | | Spain | Spain | World Olive Germplasm Bank of Marrakech |
| Lazzero di prata | MAR00367 | | Spain | Spain | World Olive Germplasm Bank of Marrakech |
| Lechin de Sevilla | MAR00243 | | Spain | Spain | World Olive Germplasm Bank of Marrakech |
| Lechin de Granada | MAR00340 | | Spain | Spain | World Olive Germplasm Bank of Marrakech |
| Lentisca-244 | MAR00244 | | Spain | Spain | World Olive Germplasm Bank of Marrakech |
| Limoncillo | MAR00341 | | Spain | Spain | World Olive Germplasm Bank of Marrakech |
| Lloron de Atarfe | MAR00245 | | Spain | Spain | World Olive Germplasm Bank of Marrakech |
| Llumeta | MAR00343 | | Spain | Spain | World Olive Germplasm Bank of Marrakech |
| Loaime | MAR00344 | | Spain | Spain | World Olive Germplasm Bank of Marrakech |
| Lucques | MAR00195 | | France | France | World Olive Germplasm Bank of Marrakech |
| Machorron | MAR00247 | | Spain | Spain | World Olive Germplasm Bank of Marrakech |
| Manzanilla Cacerena | MAR00248 | | Spain | Spain | World Olive Germplasm Bank of Marrakech |
| Manzanilla de Sevilla | MAR00251 | | Spain | Spain | World Olive Germplasm Bank of Marrakech |
| Manzanilla de Agua | MAR00345 | | Spain | Spain | World Olive Germplasm Bank of Marrakech |
| Manzanilla de Hellin | MAR00346 | | Spain | Spain | World Olive Germplasm Bank of Marrakech |
| Manzanilla de Montefrio | MAR00250 | | Spain | Spain | World Olive Germplasm Bank of Marrakech |
| Mesyaf-641 | MAR00641 | | Spain | Spain | World Olive Germplasm Bank of Marrakech |
| Mignolo Cerretano | MAR0046 | | Spain | Spain | World Olive Germplasm Bank of Marrakech |
| Minekiri | MAR00634 | | Spain | Spain | World Olive Germplasm Bank of Marrakech |
| Mollar de Cieza | MAR00348 | | Spain | Spain | World Olive Germplasm Bank of Marrakech |
| Morchione | MAR0067 | | France | France | World Olive Germplasm Bank of Marrakech |
| Morisca | MAR00254 | | Spain | Morocco | World Olive Germplasm Bank of Marrakech |
| Morona | MAR00246 | | Spain | Spain | World Olive Germplasm Bank of Marrakech |
| Morrut | MAR00350 | | Spain | Spain | World Olive Germplasm Bank of Marrakech |
| Negral de Sabinan-255 | MAR00255 | | Spain | Spain | World Olive Germplasm Bank of Marrakech |
| Negrillo Redondo | MAR00257 | | Spain | Spain | World Olive Germplasm Bank of Marrakech |
| Negrillo de Arjona | MAR00256 | | Spain | Spain | World Olive Germplasm Bank of Marrakech |
| Negrillo de Estepa | MAR00351 | | Spain | Spain | World Olive Germplasm Bank of Marrakech |
| Negrillo de Iznalloz | MAR00352 | | Spain | Spain | World Olive Germplasm Bank of Marrakech |
| Nerba | MAR00123 | | Spain | Spain | World Olive Germplasm Bank of Marrakech |
| Nevado Azul | MAR00354 | | Spain | Spain | World Olive Germplasm Bank of Marrakech |
| Nevado Basto | MAR00225 | | Spain | Spain | World Olive Germplasm Bank of Marrakech |
| Nevado Rizado | MAR00355 | | Spain | Spain | World Olive Germplasm Bank of Marrakech |
| Ocal | MAR00258 | | Spain | Spain | World Olive Germplasm Bank of Marrakech |
| Ojo de Liebre | MAR00259 | | Spain | Spain | World Olive Germplasm Bank of Marrakech |
| Olivo de Mancha Real | MAR00260 | | Spain | Spain | World Olive Germplasm Bank of Marrakech |
| Olivo di Mandanici | MAR00136 | | Syria | Spain | World Olive Germplasm Bank of Marrakech |
| Palomar | MAR00262 | | Spain | Spain | World Olive Germplasm Bank of Marrakech |
| Patronet | MAR00263 | | Spain | Spain | World Olive Germplasm Bank of Marrakech |
| Picholine | MAR00196 | | France | France | World Olive Germplasm Bank of Marrakech |
| Picholine Marocaine | MAR00540 | | Morocco | Morocco | World Olive Germplasm Bank of Marrakech |
| Pico Limon de Grazalema | MAR00265 | | Spain | Spain | World Olive Germplasm Bank of Marrakech |
| Picual | MAR00267 | | Spain | Spain | World Olive Germplasm Bank of Marrakech |
| Picudo | MAR00356 | | Spain | Spain | World Olive Germplasm Bank of Marrakech |
| Plementa Bjelica | MAR00402 | | Spain | Spain | World Olive Germplasm Bank of Marrakech |
| Puntoza | MAR00499 | | Spain | Spain | World Olive Germplasm Bank of Marrakech |
| Racimal | MAR00268 | | Spain | Spain | World Olive Germplasm Bank of Marrakech |
| Rapasayo | MAR00357 | | Spain | Spain | World Olive Germplasm Bank of Marrakech |
| Razzaio | MAR0063 | | France | France | World Olive Germplasm Bank of Marrakech |
| Rechino | MAR00269 | | Spain | Spain | World Olive Germplasm Bank of Marrakech |
| Ronde de la Menara | MAR00543 | | Morocco | Morocco | World Olive Germplasm Bank of Marrakech |
| Rossellino | MAR0060 | | Spain | Spain | World Olive Germplasm Bank of Marrakech |
| Royal de Cazorla | MAR00270 | | Spain | Spain | World Olive Germplasm Bank of Marrakech |
| Sabatera | MAR00271 | | Spain | Spain | World Olive Germplasm Bank of Marrakech |
| Salonenque | MAR00197 | | France | France | World Olive Germplasm Bank of Marrakech |
| Santa Martinenga | MAR00145 | | Syria | Spain | World Olive Germplasm Bank of Marrakech |
| Sayali | MAR00287 | | Spain | Spain | World Olive Germplasm Bank of Marrakech |
| Sevillano de Jumilla | MAR00272 | | Spain | Spain | World Olive Germplasm Bank of Marrakech |
| Sevillenca | MAR00358 | | Spain | Spain | World Olive Germplasm Bank of Marrakech |
| Sinopolese | MAR0018 | | Spain | Spain | World Olive Germplasm Bank of Marrakech |
| Storta | MAR00406 | | Spain | Spain | World Olive Germplasm Bank of Marrakech |
| Tabelout | MAR00437 | | Spain | Spain | World Olive Germplasm Bank of Marrakech |
| Tebabs | MAR00661 | | Spain | Spain | World Olive Germplasm Bank of Marrakech |
| Teffah | MAR00427 | | Spain | Spain | World Olive Germplasm Bank of Marrakech |
| Tempranillo de Yeste-274 | MAR00274 | | Spain | Spain | World Olive Germplasm Bank of Marrakech |
| Tonda Iblea | MAR0012 | | Spain | Spain | World Olive Germplasm Bank of Marrakech |
| Unkown-OT2-537 | MAR00537 | | Morocco | Morocco | World Olive Germplasm Bank of Marrakech |
| Unkown-VS1-544 | MAR00544 | | Spain | Spain | World Olive Germplasm Bank of Marrakech |
| Unkown-VS2-545 | MAR00546 | | Morocco | Morocco | World Olive Germplasm Bank of Marrakech |
| Unkown-VS2-545 | MAR00545 | | Morocco | Morocco | World Olive Germplasm Bank of Marrakech |
| Unkown-VS5-547 | MAR00547 | | Morocco | Morocco | World Olive Germplasm Bank of Marrakech |
| Uovo di Piccione | MAR00141 | | Syria | Spain | World Olive Germplasm Bank of Marrakech |
| Varudo | MAR00273 | | Spain | Spain | World Olive Germplasm Bank of Marrakech |
| Varudo-275 | MAR00275 | | Spain | Spain | World Olive Germplasm Bank of Marrakech |
| Vera | MAR00276 | | Spain | Spain | World Olive Germplasm Bank of Marrakech |
| Verdala | MAR00278 | | Spain | Spain | World Olive Germplasm Bank of Marrakech |
| Verdale | MAR00199 | | France | France | World Olive Germplasm Bank of Marrakech |
| Verdial de Badajoz | MAR00342 | | Spain | Spain | World Olive Germplasm Bank of Marrakech |
| Verdial de Huevar | MAR00213 | | Portugal | Spain | World Olive Germplasm Bank of Marrakech |
| Verdiell | MAR00279 | | Spain | Spain | World Olive Germplasm Bank of Marrakech |
| Villalonga | MAR00201 | | Portugal | Spain | World Olive Germplasm Bank of Marrakech |
| Zalmati-299 | MAR00299 | | Spain | Spain | World Olive Germplasm Bank of Marrakech |
| Zarza | MAR00280 | | Spain | Spain | World Olive Germplasm Bank of Marrakech |
| Zeletni | MAR00421 | | Spain | Spain | World Olive Germplasm Bank of Marrakech |
| Zinzala | CTO_E8_N4 | | France | France | Technical Centrer of Olive |
| Petit Ribier | CTO_P10_F06 | | France | France | Technical Centrer of Olive |
| Sabine | CTO_P10_N27 | | France | France | Technical Centrer of Olive |
| Cayet Roux | POR_P39_33_10 | | France | France | Porquerolles collection |
| Salonenque | POR_P39_33_16 | | France | France | Porquerolles collection |
| Brun | POR_P39_37_21 | | France | France | Porquerolles collection |
| Tanche | POR_P43_02_05 | | France | France | Porquerolles collection |
| Cayon | POR_P43_03_12 | | France | France | Porquerolles collection |
| Reymet | POR_P43_06_01 | | France | France | Porquerolles collection |
| Grossane | POR_P43_17_13 | | France | France | Porquerolles collection |
