# Supplementary material for: Genomic evidence of genuine wild versus admixed olive populations evolving in the same natural environments in western Mediterranean Basin
Source: PLoS One. 2024 Jan 17;19(1):e0295043. doi: 10.1371/journal.pone.0295043 (PMC10793901; doi:10.1371/journal.pone.0295043)
Supplement: S3 Table — (DOCX) [file pone.0295043.s007.docx]

**S3 Table.** **List of filters and remaining SNPs for each on genomic data of target sequencing of 561 genotypes of *O. europaea* L.**

|  | **Total SNPs remaining** |
| --- | --- |
| **Raw data** | 27275679 |
| **Remove Indel** | 24547413 |
| **Quality > 200** | 22340559 |
| **SNP Biallelic only** | 21036126 |
| **Min mean depth 8** | 958648 |
| **Max mean depth 400** | 957569 |
| **SNP cluster (3,10)** | 383904 |
| **Min depth 8** | 383904 |
| **Missing data Site > 0.15** | 155063 |
| **heterozygoty > 0.85** | 154675 |
| **Minor allele count 1** | 142060 |
