# Supplementary material for: Genomic evidence of genuine wild versus admixed olive populations evolving in the same natural environments in western Mediterranean Basin
Source: PLoS One. 2024 Jan 17;19(1):e0295043. doi: 10.1371/journal.pone.0295043 (PMC10793901; doi:10.1371/journal.pone.0295043)
Supplement: S4 Table — (DOCX) [file pone.0295043.s008.docx]

**S4 Table.** **List of individuals of *O. europaea* L. removed from genomic data set because of missing data >0.2**

| **Sample ID removed** | **Type** |
| --- | --- |
| Manzanilla_de_Abla | Cultivated |
| OES_E13_03 | Wild |
| OES_E15_08 | Wild |
| OES_E15_12 | Wild |
| OES_E16_05 | Wild |
| OES_E16_10 | Wild |
| OES_E16_13 | Wild |
| OES_E17_08 | Wild |
| OES_E18_02 | Wild |
| OES_E18_04 | Wild |
| OES_E18_11 | Wild |
| OES_E19_12 | Wild |
| OES_E20_02 | Wild |
| OES_F02_01 | Wild |
| OES_F02_15 | Wild |
| OES_F07_08 | Wild |
| OES_F07_10 | Wild |
| OES_F10_05 | Wild |
| OES_F10_13 | Wild |
| OES_F11_04 | Wild |
| OES_M21_03 | Wild |
| OES_M21_13 | Wild |
| OES_M23_03 | Wild |
| OES_M23_05 | Wild |
| OES_M23_07 | Wild |
| OES_M23_09 | Wild |
| OES_M23_10 | Wild |
| OES_M23_11 | Wild |
| OES_M23_13 | Wild |
| OES_M23_14 | Wild |
| OES_M23_15 | Wild |
| OES_M28_10 | Wild |
| OES_M29_04 | Wild |
| OST072 | Wild |
| OST082 | Wild |
