# Supplementary material for: Genomic evidence of genuine wild versus admixed olive populations evolving in the same natural environments in western Mediterranean Basin
Source: PLoS One. 2024 Jan 17;19(1):e0295043. doi: 10.1371/journal.pone.0295043 (PMC10793901; doi:10.1371/journal.pone.0295043)
Supplement: S6 Table — (DOCX) [file pone.0295043.s010.docx]

**S6 Table. List of cultivated accessions of *O. europaea* L. and their assignment to genetic cluster (C0, C1, C3 or C4) according to sNMF assignation at K = 4.**

| **Accession names** | **Cluster assignation** |
| --- | --- |
| Unkown-VS2-545 | C0 |
| Acebuchera | C4 |
| Aglandau | C1 |
| Alameno Blanco | C4 |
| Alameno de Montilla | C0 |
| Amargoso | C1 |
| Arbequina | C3 |
| Azul | C0 |
| Berri Meslal-397 | C0 |
| Berri Meslal-532 | C0 |
| Bical | C4 |
| Blanqueta | C3 |
| Bolvino | C0 |
| Borriolenca | C0 |
| Bouchouika | C4 |
| Bouteillan | C1 |
| Zinzala | C3 |
| Petit Ribier | C3 |
| Sabine | C0 |
| Callosina | C4 |
| Canivano Negro | C4 |
| Carrasqueno de Jumilla | C4 |
| Carrasquillo | C4 |
| Cayon | C1 |
| Cerezuela | C4 |
| Changlot Real | C0 |
| Chorruo | C4 |
| Cirujal | C0 |
| Corbella | C0 |
| Cornezuelo de Jaen | C0 |
| Cornicabra | C4 |
| Cucca | C0 |
| Dolce di Rossano | C0 |
| Dressi | C0 |
| Dulzal | C4 |
| El Lewa | C1 |
| Empeltre | C0 |
| Enagua de Arenas | C4 |
| Escarabajuelo de Posadas | C4 |
| Escarabajuelo de Úbeda | C0 |
| Farga | C0 |
| Frantoio | C3 |
| Fulla de Salze | C4 |
| Gentile di chieti | C3 |
| Gordal Sevillana | C1 |
| Gordal de Granada | C4 |
| Grappolo | C0 |
| Grossane-194 | C1 |
| Habichuelero de Grazalema | C4 |
| Hojiblanca | C4 |
| Idleb | C1 |
| Jabaluna | C0 |
| Jaropo | C4 |
| Khashabi-631 | C1 |
| Lastrino | C0 |
| Lazzero di prata | C3 |
| Lechin de Sevilla | C0 |
| Lechin de Granada | C0 |
| Lentisca-244 | C0 |
| Limoncillo | C4 |
| Lloron de Atarfe | C4 |
| Llumeta | C0 |
| Loaime | C4 |
| Lucques | C1 |
| Machorron | C4 |
| Manzanilla Cacerena | C4 |
| Manzanilla de Sevilla | C4 |
| Manzanilla de Agua | C4 |
| Manzanilla de Hellin | C4 |
| Manzanilla de Montefrio | C4 |
| Mesyaf-641 | C1 |
| Mignolo Cerretano | C3 |
| Minekiri | C1 |
| Mollar de Cieza | C4 |
| Morchione | C0 |
| Morisca | C4 |
| Morona | C4 |
| Morrut | C0 |
| Negral de Sabinan-255 | C1 |
| Negrillo Redondo | C4 |
| Negrillo de Arjona | C4 |
| Negrillo de Estepa | C4 |
| Negrillo de Iznalloz | C4 |
| Nerba | C1 |
| Nevado Azul | C4 |
| Nevado Basto | C4 |
| Nevado Rizado | C4 |
| Ocal | C4 |
| Ojo de Liebre | C4 |
| Olivo de Mancha Real | C4 |
| Olivo di Mandanici | C3 |
| Cayet Roux | C0 |
| Salonenque | C1 |
| Brun | C1 |
| Tanche | C1 |
| Cayon | C0 |
| Reymet | C3 |
| Grossane | C1 |
| Palomar | C0 |
| Patronet | C0 |
| Picholine | C0 |
| Picholine Marocaine | C4 |
| Pico Limon de Grazalema | C4 |
| Picual | C4 |
| Picudo | C4 |
| Plementa Bjelica | C0 |
| Puntoza | C0 |
| Racimal | C4 |
| Rapasayo | C0 |
| Razzaio | C3 |
| Rechino | C4 |
| Ronde de la Menara | C4 |
| Rossellino | C3 |
| Royal de Cazorla | C4 |
| Sabatera | C0 |
| Salonenque | C1 |
| Santa Martinenga | C0 |
| Sayali | C0 |
| Sevillano de Jumilla | C4 |
| Sevillenca | C0 |
| Sinopolese | C0 |
| Storta | C0 |
| Tabelout | C3 |
| Tebabs | C1 |
| Teffah | C1 |
| Tempranillo de Yeste-274 | C4 |
| Tonda Iblea | C1 |
| Unkown-OT2-537 | C3 |
| Unkown-VS1-544 | C0 |
| Unkown-VS2-545 | C0 |
| Unkown-VS2-545 | C0 |
| Unkown-VS5-547 | C0 |
| Uovo di Piccione | C1 |
| Varudo | C4 |
| Varudo-275 | C4 |
| Vera | C3 |
| Verdala | C4 |
| Verdale | C1 |
| Verdial de Badajoz | C4 |
| Verdial de Huevar | C4 |
| Verdiell | C0 |
| Villalonga | C0 |
| Zalmati-299 | C3 |
| Zarza | C0 |
| Zeletni | C0 |
