# Supplementary material for: Genomic evidence of genuine wild versus admixed olive populations evolving in the same natural environments in western Mediterranean Basin
Source: PLoS One. 2024 Jan 17;19(1):e0295043. doi: 10.1371/journal.pone.0295043 (PMC10793901; doi:10.1371/journal.pone.0295043)
Supplement: S7 Table — (DOCX) [file pone.0295043.s011.docx]

**S7 Table. Descriptive data of depth and enrichment rate of *O. europaea* L. using target sequencing method versus whole genome sequencing method.**

| **Sample ID** | **Mean depth whole genome** | **Mean depth target sequencing** | **Enrichment rate** |
| --- | --- | --- | --- |
| OES_E13_09 | 1.21 | 40.40 | 33.27 |
| OES_F10_03 | 1.27 | 34.72 | 27.31 |
| Picholine | 0.77 | 18.20 | 23.59 |
| Picholine_Marocaine | 1.27 | 67.20 | 52.88 |
| Mean_all | 1.13 | 40.13 | 34.26 |
